# Predicting Density and Elucidating the Thermodynamic Drivers of Viscosity in Carboxy-Functionalized Imidazolium Ionic Liquids

**DOI:** 10.3390/molecules31142495

**Published:** 2026-07-17

**Authors:** Nikolett Cakó Bagány, Sanja Armaković, Stevan Armaković, Bojan Šarac, Sanja Belić, Romana Cerc-Korošec, Marija Bešter-Rogač, Slobodan Gadžurić

**Affiliations:** 1 Faculty of Science, University of Novi Sad, Trg Dositeja Obradovića 3, 21000 Novi Sad, Serbia; nikolet.cakobaganj@dh.uns.ac.rs (N.C.B.); sanja.armakovic@dh.uns.ac.rs (S.A.); stevan.armakovic@df.uns.ac.rs (S.A.); sanja.belic@dh.uns.ac.rs (S.B.); 2 Faculty of Chemistry and Chemical Technology, University of Ljubljana, Večna pot 113, 1000 Ljubljana, Slovenia; bojan.sarac@fkkt.uni-lj.si (B.Š.); romana.cerc-korosec@fkkt.uni-lj.si (R.C.-K.); marija.bester@fkkt.uni-lj.si (M.B.-R.)

**Keywords:** carboxy-functionalized imidazolium ion, density functional theory, symmetry-adapted perturbation theory, machine learning, ion–ion interactions, ionic liquid smart design

## Abstract

The rational design of ionic liquids (ILs) is often hindered when promising candidates, such as carboxy-functionalized imidazolium chlorides, exhibit properties like extreme viscosity that preclude direct experimental measurement. In this study, we synthesized a series of these ILs and addressed this “experimental gap” with a combined computational strategy. For the few liquids accessible to measurement, we obtained density, viscosity, and conductivity data. For the majority, we turned to atomistic modeling and machine learning. Symmetry-adapted perturbation theory (SAPT2) energy decomposition uncovered the dominance of electrostatic interactions in governing viscosity, an insight obscured by total binding energies from DFT. In addition, a recently developed machine learning model, named IonIL-IM-D1, predicted the density of [C_2_COOHeim][Cl] with an error of less than 1% upon validation, though experimental verification for the solid candidates was not possible. This predictive framework was extended to propose and evaluate new IL candidates, offering a complementary strategy for exploring macroscopic behavior when direct experimental measurements are not feasible.

## 1. Introduction

Ionic liquids have attracted considerable attention over the past two decades due to their unique and tunable physicochemical properties. Among the many families of ILs, imidazolium-based ionic liquids are particularly noteworthy due to their straightforward synthesis, structural versatility, and favorable physicochemical behavior [1,2,3]. Functionalization of the imidazolium ion, particularly with carboxylic acid groups, presents a powerful strategy for enhancing properties like hydrogen bonding capacity, polarity, and water solubility [4,5], making it increasingly relevant for applications in biomolecule solubilization, pharmaceutical formulation, and CO_2_ capture [6,7,8].

However, this very tunability introduces a significant characterization challenge. Promising ILs with strong intermolecular interactions often exhibit extreme viscosities or are solid at room temperature, rendering them inaccessible to routine experimental measurement of key properties like density and viscosity. This creates a critical gap in structure–property relationships, hindering the rational design of next-generation functionalized ILs.

Atomistic modeling techniques are indispensable for bridging this experimental gap. Density functional theory provides detailed insights into molecular geometry, electronic structure, and thermodynamic stability [9,10,11], while symmetry-adapted perturbation theory offers a rigorous, quantitative decomposition of intermolecular forces into electrostatic, induction, dispersion, and exchange-repulsion components [12,13,14]. These methods are vital for elucidating the fundamental ion–ion interactions that dictate macroscopic IL behavior [15,16,17]. The application of atomistic calculations in the present work was further motivated by recent studies demonstrating the value of high-level quantum-mechanical methods for investigating ionic liquids beyond conventional electronic structure calculations. In particular, SAPT-based energy decomposition has provided detailed insight into the balance of electrostatic, exchange-repulsion, induction, and dispersion interactions governing ion-pair association in both conventional imidazolium-based and magnetic ionic liquids [18,19]. Moreover, SAPT-derived interaction energy components have proven instrumental in the development of physically motivated polarizable force fields, significantly improving the prediction of structural, thermodynamic, and transport properties across diverse ionic-liquid families [20,21]. These studies collectively highlight the capability of SAPT not only to quantify intermolecular interactions but also to establish physically meaningful links between molecular-level interactions and macroscopic behavior, providing a strong rationale for its application to functionalized ionic liquids.

However, high-level quantum calculations become prohibitively expensive when screening large numbers of potential candidates, necessitating complementary data-driven approaches. Machine learning has emerged as a powerful tool for the rapid prediction of material properties, capable of identifying patterns in quantum-chemical or experimental data to accurately estimate properties such as density, viscosity, melting point, and electrochemical window for large libraries of candidate compounds at a fraction of the computational cost of traditional simulations [22,23,24,25]. In the context of ionic liquids, ML can transform descriptive understanding into a predictive framework for IL smart design.

In this study, we address the experimental inaccessibility of a series of highly cohesive carboxy-functionalized imidazolium-based chloride ionic liquids (ILs)—specifically, 1-carboxymethyl-3-methylimidazolium chloride ([C_1_COOHmim][Cl]), 1-carboxyethyl-3-methylimidazolium chloride ([C_2_COOHmim][Cl]), 1-carboxymethyl-3-ethylimidazolium chloride ([C_1_COOHeim][Cl]), 1-carboxyethyl-3-ethylimidazolium chloride ([C_2_COOHeim][Cl]), and 1-carboxyethyl-3-butylimidazolium chloride ([C_2_COOHbim][Cl])—through an integrated computational strategy. We synthesized a series of these ILs, and where direct measurement was possible, we used the data for validation. For the majority of compounds where experiments failed, we employed a synergistic approach of density functional theory (DFT) and symmetry-adapted perturbation theory (SAPT2) calculations to quantify and decompose ion-pair interactions, revealing the molecular-level origins of their behavior, and an ML model to predict the densities of all synthesized ILs and propose new candidates. Thus, the novelty of the present work lies in the combined application of SAPT2 energy decomposition and machine learning to elucidate the molecular drivers of extreme viscosity in carboxy-functionalized ILs, the experimental characterization of four new ILs ([C_1_COOHmim][Cl], [C_1_COOHeim][Cl], [C_2_COOHeim][Cl], and [C_2_COOHbim][Cl]), and the demonstration of a predictive framework for proposing and evaluating new IL candidates with tailored properties, extending beyond the single compound reported in the ML model development in [26] and in our previous work [27].

## 2. Results and Discussion

The extreme viscosities and solid-state natures constrained the experimental characterization of the synthesized carboxy-functionalized ILs at room temperature. While density, viscosity, and conductivity data were successfully obtained for [C_2_COOHeim][Cl] and viscosity for [C_2_COOHbim][Cl], a comprehensive understanding of the structure–property relationships required a combined computational strategy. This section first presents the available experimental data, highlighting key trends and anomalies, and then employs density functional theory, symmetry-adapted perturbation theory, and machine learning to elucidate the molecular origins of these properties and predict them for the inaccessible compounds.

### 2.1. Experimental Physicochemical Properties

**Figure 1:**
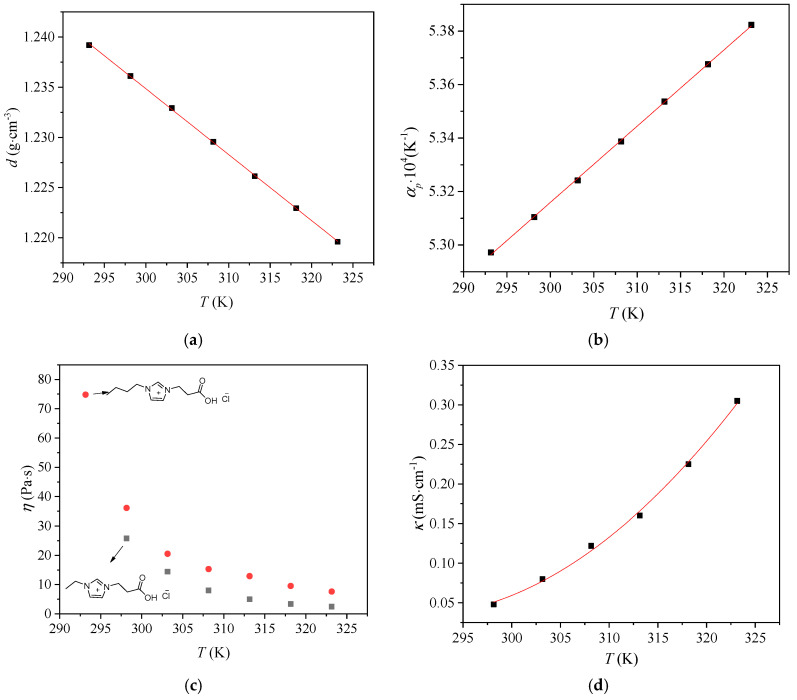
(**a**) Variation in [C_2_COOHeim][Cl] density (*d*) with temperature (*T*), (**b**) variation in thermal expansion coefficient (*α_p_*) of [C_2_COOHeim][Cl] with temperature (*T*), (**c**) effect of temperature (*T*) and alkyl chain length on the viscosity (*η*) of ionic liquids: [C_2_COOHeim][Cl] (black squares), [C_2_COOHbim][Cl] (red circles), and (**d**) variation in specific conductivity (*κ*) of [C_2_COOHeim][Cl] with temperature (*T*).

Density measurements were successfully performed for [C_2_COOHeim][Cl] across a temperature range from *T* = 293.15 to 323.15 K at atmospheric pressure (Table S1). As expected, the density decreased linearly with increasing temperature (Figure 1a) as a result of enhanced molecular motion and the thermal expansion of free volume within the liquid.

Using the slope derived from the relationship between the change in ionic liquid density and temperature, the isobaric thermal expansion coefficient (*α_p_*) was determined:

(1) $$d=b_{o}+b\cdot T$$

(2) $$b=\frac{\partial d}{\partial T}$$

(3) $$\alpha_{p}=-\frac{1}{d}\cdot b$$

The values of *α_p_* reflect how a substance’s shape, structure, or volume varies in response to temperature changes. The obtained values presented in Table S1 and illustrated in Figure 1b showed a steady linear increase with temperature, confirming the expected reduction in packing efficiency at higher thermal energy.

Viscosity measurements in the temperature range from *T* = 293.15 to 323.15 K revealed a structure–property relationship consistent with previous observations in imidazolium-based ILs (Table S2). Both [C_2_COOHeim][Cl] and [C_2_COOHbim][Cl] exhibited a strong negative temperature dependence, with viscosity decreasing as temperature increased due to weakened ion–ion interactions and enhanced mobility (Figure 1c). However, in agreement with the well-established trend in many imidazolium ILs where longer alkyl chains increase viscosity, the IL with the butyl chain ([C_2_COOHbim][Cl]) was markedly more viscous than its ethyl-chain analogue ([C_2_COOHeim][Cl]). This result is consistent with the expected behavior, where the increased steric bulk and hydrophobic nature of the butyl group enhance van der Waals interactions and hinder molecular mobility, thereby increasing viscosity. The extended alkyl chain may also contribute to stronger dispersion forces and more significant molecular entanglement, leading to greater resistance to flow. The Brookfield DV II+ Pro viscometer has a maximum measurable viscosity of approximately 10^6^ cP (1000 Pa·s) with the SC4-18 spindle used. The remaining ILs, [C_1_COOHmim][Cl], [C_1_COOHeim][Cl], and [C_2_COOHmim][Cl], exhibited viscosities exceeding this limit at room temperature, consistent with their solid or extremely viscous state.

Consistent with the viscosity results, the electrical conductivity of [C_2_COOHeim][Cl] increased with temperature (Table S1, Figure 1d). The observed inverse relationship between conductivity and viscosity in [C_2_COOHeim][Cl] is consistent with the general expectation that ILs with higher viscosity, as inferred for the unmeasurable compounds from their stronger electrostatic interactions, would exhibit lower conductivity. However, direct confirmation requires future measurements under conditions where these highly viscous ILs become fluid.

**Figure 2:**
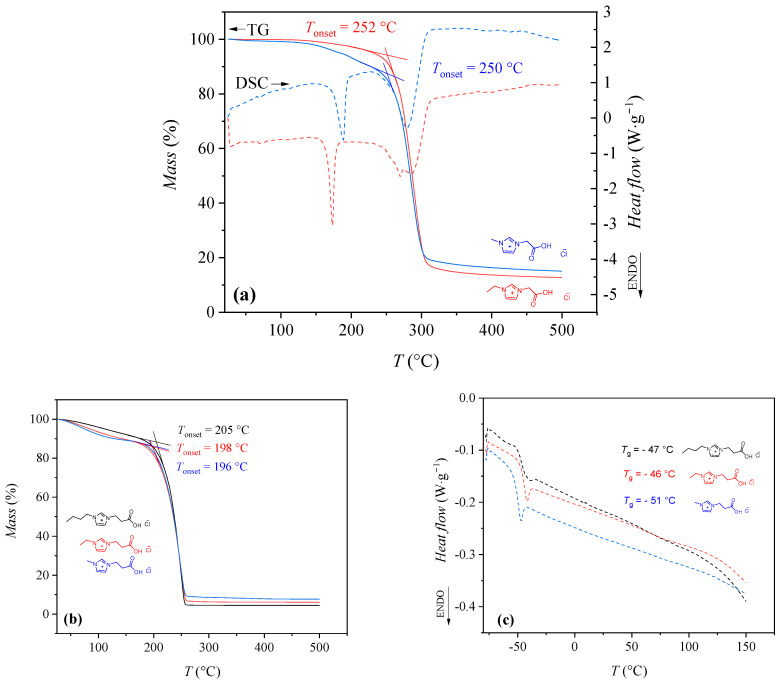
(**a**) TG and DSC curves of [C_1_COOHmim][Cl] (blue) and [C_1_COOHeim][Cl] (red) ionic liquids; (**b**) TG curves of [C_2_COOHbim][Cl] (black), [C_2_COOHeim][Cl] (red), and [C_2_COOHmim][Cl] (blue) (**c**) DSC curves of [C_2_COOHbim][Cl] (black), [C_2_COOHeim][Cl] (red), and [C_2_COOHmim][Cl] (blue).

Thermogravimetric and DSC analyses confirmed that all synthesized ILs possess high thermal stability, with decomposition onset temperatures exceeding 190 °C (Figure 2). The solid-state ILs ([C_1_COOHmim][Cl] and [C_1_COOHeim][Cl] shown in Figure 2a) displayed clear melting endotherms before decomposition; the onset is 178 °C for the first and 167 °C for the second, respectively. Their thermal stability is higher compared to liquid ILs; the onset decomposition temperature is approximately 250 °C for the solid and about 200 °C for the liquid ILs. The liquid ILs showed glass transition events at a low temperature and no sharp melting points, characteristic of their amorphous nature. In the 1-carboxyethyl samples, the thermal stability depends slightly on the length of the alkyl chain attached to the second imidazolium nitrogen atom. The longer the chain, the higher the thermal stability, indicating that the hydrophobic interactions of these chains also have a small influence on the thermal resilience (Figure 2b). Although there is a greater difference in thermal stability between [C_2_COOHbim][Cl], which is the most thermally stable, and the other two, [C_2_COOHeim][Cl] and [C_2_COOHmim][Cl], the glass transition of the latter two is very similar (mean value −46.5 °C), and that of the shortest (methyl) group bonded to the imidazolium ring is the lowest (−51 °C) as shown in Figure 2c. One may assume that, besides thermal stability, the length of the chain mentioned also affects molecular mobility.

### 2.2. Molecular-Level Insights from Atomistic Calculations

To decipher the molecular mechanisms behind these macroscopic properties, particularly the unexpected viscosity trend, we quantified ion-pair interactions using DFT and SAPT2. The total ion-pair binding energies (IPBEs) calculated at the r^2^SCAN-3c level of theory are summarized in Table 1. While the values were largely similar (approximately −102 to −104 kcal·mol^−1^) for most ILs, they failed to provide a clear correlation with the experimental trends. For instance, [C_2_COOHmim][Cl] had a weaker IPBE than [C_2_COOHeim][Cl] but was too viscous to measure, indicating that the total binding energy is an insufficient descriptor for bulk properties like viscosity. This disparity underscores that the total binding energy of an isolated ion pair is only a first-order approximation for bulk flow resistance; higher-order packing effects and the balance between attractive and repulsive interactions must be considered.

The discrepancy was resolved by decomposing the interaction energy using SAPT2 (Table 2). The SAPT2 analysis revealed that the electrostatic (EL) component is the overwhelmingly dominant attractive force across all ILs, whereas the dispersion contribution remained comparatively small.

Previous studies by Izgorodina et al. on the interaction energy components of ionic liquids have demonstrated that, in ionic-liquid families containing large, weakly coordinating, and highly polarizable ions, variations in dispersion interactions can correlate with transport properties, despite electrostatics remaining the dominant contribution to the total interaction energy [15,16,17].

In contrast, the present study focuses exclusively on chloride-based ionic liquids, where the compact chloride anion possesses a highly localized negative charge and strong hydrogen-bond accepting ability. Under these conditions, electrostatic interactions are expected to play a comparatively greater role in determining ion-pair cohesion than the relatively small variations in dispersion.

While the limited experimental viscosity dataset does not permit establishing a rigorous quantitative correlation between the electrostatic interaction energy and viscosity, the SAPT2 results provide a physically meaningful molecular interpretation of the experimentally observed behavior. In particular, ionic liquids exhibiting the strongest electrostatic interactions, namely [C_1_COOHmim][Cl], [C_1_COOHeim][Cl], and [C_2_COOHmim][Cl], are either solid at room temperature or possess viscosities exceeding the experimental measurement range, indicating exceptionally strong cohesive interactions. In contrast, the weaker electrostatic interactions calculated for [C_2_COOHeim][Cl] and [C_2_COOHbim][Cl] are consistent with these compounds remaining sufficiently fluid for quantitative viscosity measurements.

The comparison between the last-mentioned two liquid ionic liquids further indicates that electrostatics alone cannot fully explain the observed viscosity difference. Although their electrostatic components differ only slightly, [C_2_COOHbim][Cl] exhibits a substantially higher viscosity than [C_2_COOHeim][Cl], suggesting that secondary factors, including steric effects and enhanced dispersion interactions associated with the longer butyl substituent, also contribute to the final macroscopic behavior. Therefore, within the present family of carboxy-functionalized chloride ionic liquids, electrostatic interactions appear to govern the overall cohesive strength, while dispersion and steric effects provide an additional level of fine-tuning of the transport properties.

It should also be noted that the present SAPT2 calculations were performed for isolated gas-phase ion pairs and therefore do not explicitly account for many-body and long-range structural effects present in the condensed phase. Consequently, the calculated interaction energies are intended to provide a physically meaningful molecular interpretation of the observed experimental trends rather than a direct quantitative description of bulk viscosity.

### 2.3. Predictive Power of Machine Learning and Smart Design

Having established the molecular foundations of viscosity through DFT and SAPT2 calculations, we employed a machine learning (IonIL-IM-D1) model [26] to predict density, a property difficult to measure for most of our synthesized ILs due to their extreme viscosity or solid-state nature. The model’s predictions, together with the used descriptors (molecular weight, number of atoms, and AlogP) for the synthesized ILs, are presented in Table 3. IonIL-IM-D1 model was also applied for [C_2_COOHeim][Cl], where the predicted density (1.23519 g·cm^−3^) was in excellent agreement (difference < 1%) with the experimental value (1.23611 g·cm^−3^). For the other ILs, the model predicted high densities (1.25–1.27 g·cm^−3^), consistent with their strong cohesive forces observed in SAPT2 calculations (Table 2).

A consolidated view of the data (Table 4) reveals a coherent picture linking molecular interactions to macroscopic behavior. ILs with stronger SAPT2 electrostatic components and higher predicted densities, such as the solid [C_1_COOHmim][Cl] and [C_1_COOHeim][Cl], also exhibit higher thermal decomposition temperatures (~250 °C). This confirms that the electrostatic cohesion governing ion packing and flow resistance also enhances thermal resilience. In contrast, the liquid ILs with slightly weaker electrostatic interactions have correspondingly lower decomposition onsets (~196–205 °C). This consistent trend across density, viscosity, and thermal stability underscores the dominance of electrostatic forces in determining the key physicochemical properties of these carboxy-functionalized ILs.

To demonstrate the practical utility of this integrated framework, we proposed five new IL candidates (Table 5) with systematic structural modifications. The selection of these specific candidates was guided by clear design logic aimed at testing the predictive capability of our computational framework and exploring structure–property relationships beyond the experimentally accessible range.

1. Extending the carboxy-alkyl chain, testing whether further elongation of the carboxy-bearing side chain continues to reduce electrostatic cohesion, as suggested by the trend from [C_1_COOHmim][Cl] to [C_2_COOHmim][Cl] (Table 2), potentially yielding an IL with lower viscosity that remains liquid at room temperature while retaining the functional carboxyl group.

2. Introducing additional hydrogen bonding sites by the introduction of a hydroxyl group adjacent to the carboxyl group on the side chain or on the N-alkyl substituent. These modifications are designed to test whether additional hydrogen bond donors increase ion-pair cohesion beyond what is observed for the carboxyl group alone, potentially leading to enhanced properties for applications requiring strong intermolecular interactions (e.g., CO_2_ capture, biomolecule stabilization).

3. Exploring long alkyl chain effects: The candidates [C_9_COOHmim][Cl] (1-carboxynonyl-3-methylimidazolium chloride) and [C_11_COOHmim][Cl] (1-carboxyundecyl-3-methylimidazolium chloride) feature substantially extended N-alkyl chains, representing a transition to long-chain amphiphilic ILs. The large jump from short to long alkyl chains is intentional: it allows exploration of the regime where steric effects, van der Waals interactions, and hydrophobic associations are expected to dominate over electrostatic interactions, potentially yielding ILs with significantly lower densities and viscosities. This design tests the hypothesis, derived from our SAPT2 results, that weakening the electrostatic component through increased steric hindrance can dramatically reduce viscosity while introducing amphiphilic character beneficial for applications such as surfactant-assisted extractions or as phase-change materials.

**Figure 3:**
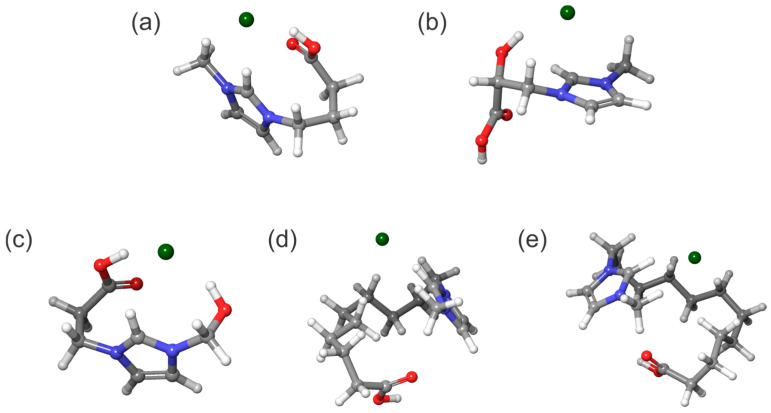
Optimized geometries of proposed ILs: (**a**) [C_3_COOHmim][Cl], (**b**) [C_2_(OH)COOHmim][Cl], (**c**) [C_2_COOHm(OH)im][Cl], (**d**) [C_9_COOHmim][Cl], and (**e**) [C_11_COOHmim][Cl].

For all proposed candidates, global minima obtained via GOAT and successive optimizations at the r^2^SCAN-3c level of theory are provided in Figure 3. Their DFT-calculated ion-pair binding energies (IPBEs) and SAPT2-decomposed interaction energies are summarized in Table 6 and Table 7, respectively.

Using an IonIL-IM-D1 model, we estimated their physical properties prior to synthesis, helping to prioritize candidates with desirable density or viscosity profiles. This approach supports the rational design of ILs and highlights how computational tools can enhance experimental planning, especially in cases where direct measurements are limited by instrumental constraints.

The results reveal crucial structure–property relationships for the proposed ILs. The introduction of an additional hydroxyl group in [C_2_COOHm(OH)im][Cl] leads to a profound strengthening of the electrostatic component (−131.36 kcal·mol^−1^), which is the most negative among all synthesized and proposed ILs investigated in this study. This suggests that this candidate might exhibit extremely high viscosity, consistent with the strong hydrogen-bonding network facilitated by the hydroxymethyl substituent. Similarly, [C_2_(OH)COOHmim][Cl] shows a substantially enhanced electrostatic component (−125.93 kcal·mol^−1^) compared to its non-hydroxylated analogue [C_2_COOHmim][Cl] (−120.22 kcal·mol^−1^), confirming that additional hydrogen bond donors significantly increase ion-pair cohesion.

In contrast, extending the N-alkyl chain to nonyl and undecyl ([C_9_COOHmim][Cl] and [C_11_COOHmim][Cl]) dramatically weakens electrostatic interactions (EL components of −98.16 and −98.14 kcal·mol^−1^, respectively, compared to −120.22 kcal·mol^−1^ for [C_2_COOHmim][Cl]). The total SAPT2 interaction energies of approximately −86 kcal/mol for these long-chain ILs represent a substantial reduction in ion-pair cohesion compared to the short-chain analogues. This weakening is attributed to increased steric hindrance between the bulky alkyl chains and the chloride anion, which disrupts optimal electrostatic alignment. Based on these findings, we hypothesize that these long-chain candidates would exhibit substantially lower viscosities than their short-chain counterparts, making them attractive targets for applications requiring efficient mass transport.

This trend is mirrored in the ML-predicted densities (Table 8), which decrease substantially for the long-chain ILs. The predicted densities for the proposed ionic liquids, obtained using the IonIL-IM-D1 model, reveal a consistent trend with the previously discussed SAPT2 and IPBE results. In the case of [C_3_COOHmim][Cl], [C_2_(OH)COOHmim][Cl], and [C_2_COOHm(OH)im][Cl], which showed strong electrostatic interaction components and high total ion-pair binding energies, relatively high predicted densities in the range of 1.23–1.30 g·cm^−3^ were obtained. This is consistent with their tighter ion packing and reduced free volume, as implied by their stronger cohesive forces. In contrast, the model predicts significantly lower densities for [C_9_COOHmim][Cl] (1.099 g·cm^−3^) and [C_11_COOHmim][Cl] (1.073 g·cm^−3^), positioning them as promising targets for applications where lower density and potentially lower viscosity are advantageous.

A consolidated view of the data (Table 4) reveals a coherent picture linking molecular interactions to macroscopic behavior. ILs with stronger SAPT2 electrostatic components and higher predicted densities, such as the solid [C_1_COOHmim][Cl] and [C_1_COOHeim][Cl], also exhibit higher thermal decomposition temperatures (~250 °C). This confirms that the electrostatic cohesion governing ion packing and flow resistance also enhances thermal resilience. In contrast, the liquid ILs with slightly weaker electrostatic interactions have correspondingly lower decomposition onsets (~196–205 °C). This consistent trend across density, viscosity, and thermal stability underscores the dominance of electrostatic forces in determining the key physicochemical properties of these carboxy-functionalized ILs, while also revealing the important role of alkyl chain effects in modulating these properties.

## 3. Materials and Methods

### 3.1. Synthesis of Ionic Liquids

All reagents used in the synthesis of ionic liquids were commercially available, purchased from Sigma Aldrich (St. Louis, MO, USA), and used without further purification. For the synthesis, the appropriate alkylated imidazole (methylimidazole, ethylimidazole, or butylimidazole) was weighed and dissolved in acetonitrile. Subsequently, chloroacetic or chloropropionic acid was added in a ten percent molar excess. The mixture of the corresponding component and solvent was refluxed for 72 h at a temperature of 343.15 K. Upon completion of the reaction, two distinct layers formed: the upper layer contained the unreacted components dissolved in acetonitrile, while the lower layer contained the ionic liquid. In cases where the resulting ionic liquids were in a solid state at room temperature, a white powdery substance precipitated in the lower layer. Acetonitrile was removed using a rotary vacuum evaporator at 343.15 K.

The resulting ionic liquids were purified by liquid-liquid extraction or solid-liquid separation using a small amount of ethyl acetate. This process was repeated until a clear upper layer was obtained. The following ionic liquids were obtained as green/yellowish liquids at room temperature: [C_2_COOHmim][Cl], [C_2_COOHeim][Cl], and [C_2_COOHbim][Cl]. In contrast, [C_1_COOHmim][Cl] and [C_1_COOHeim][Cl] were obtained as white crystalline products. Prior to the physicochemical measurements, all ionic liquids were dried under high vacuum (10^−3^ mbar) at 333.15 K for 48 h and stored in desiccators over P_2_O_5_ until used to minimize moisture uptake. The water content of the studied ionic liquids was determined by Karl Fischer titration using a Metrohm 831 Karl Fischer coulometer and was found to be below 0.05 wt% for all samples. The purity of the synthesized ionic liquids was confirmed by NMR spectroscopy, which indicated no detectable residual starting materials or solvents within the detection limits of the method.

**Figure 4:**
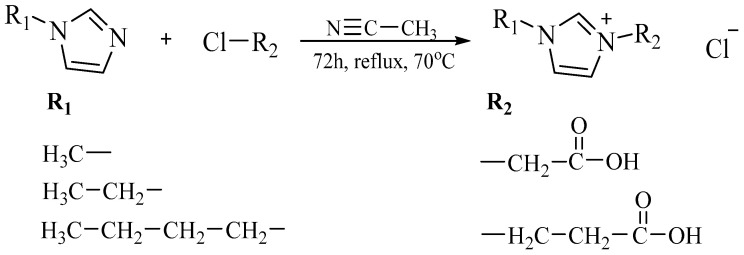
Ionic liquids: synthetic path.

The structures of the synthesized ionic liquids were confirmed by both NMR and IR spectroscopy, as shown in the corresponding figures with appropriate signal assignments (Figures S1–S4 for NMR and Figures S5–S8 for FTIR in the Supplementary Materials of this work for all compounds, except for [C_2_COOHmim][Cl], which is presented in our previous publication [27]). The detailed synthesis procedure is shown in Figure 4. Chemical structures, chemical names, abbreviations, and purification methods of the studied ILs are tabulated in Table 9.

### 3.2. Density, Viscosity, Electrical Conductivity, and Thermogravimetric Measurements

Density measurements of the synthesized ionic liquid were performed at atmospheric pressure (*p* = 0.1 MPa) using a Rudolph Research Analytical DDM 2911 vibrating-tube densimeter (Hackettstown, NJ, USA). Measurements were conducted over the temperature range *T* = 293.15–323.15 K, with an accuracy of ±0.00005 g·cm^−3^. The repeatability of the measurements was within 0.01%, and the standard uncertainty did not exceed 3 · 10^−4^ g·cm^−3^. Before each measurement, the instrument was calibrated at 293.15 K under atmospheric pressure using triple-distilled ultra-pure water and air. The densimeter is equipped with a built-in Peltier thermostat, ensuring a relative standard temperature uncertainty of less than 0.015 K. Any viscosity-related density deviations were automatically corrected. Each reported density represents an average of at least five measurements at the specified temperature. Approximately 1 cm^3^ of sample was used for each density determination. The device also contains an integrated moisture-absorbent material to minimize water interference.

The viscosity of the investigated ionic liquids was determined using a Brookfield DV II+ Pro viscometer (AMETEK Brookfield, Middleboro, MA, USA) equipped with a thermostat, keeping temperature stability within ±0.01 K. Approximately 15 cm^3^ of pure ionic liquid was used for each measurement. An SC4-18 spindle was immersed in the sample, and the rotation speed (RPM) was adjusted to ensure an appropriate torque range. The viscometer chamber, designed by the manufacturer, includes a protective compartment to prevent moisture interference. Calibration of the viscometer was performed using certified standard viscosity fluids covering the full range of viscosities observed in the studied ionic liquids. Measurements were carried out over a *T* = 293.15 to 323.15 K temperature range with rotation speeds varying from 0.2 to 2 RPM. Each reported viscosity value represents the average of three independent measurements, with the estimated relative standard uncertainty of approximately 0.02.

Rheological measurements of pure 1-carboxyethyl-3-butylimidazolium chloride were carried out using rheometer MCR 302 (Anton Paar GmbH, Graz, Austria) equipped with a cone-plate geometry (50 mm diameter, 1° cone angle). Measurements were conducted under a dry air atmosphere to minimize moisture uptake. Viscosity was recorded over a shear rate range of 10 to 1000 s^−1^ at temperatures from 278.15 K to 333.15 K, in 5 K increments. Prior to each measurement, the sample was equilibrated at the target temperature for 3 min to ensure thermal stability. To determine the zero-shear viscosity (*η*_0_), the experimental flow curves were fitted using the simplified Carreau model [28]:

(4) $$\eta \left(\gamma \right)=\eta_{o}{\left[1+{\left(\lambda \gamma \right)}^{2}\right]}^{\frac{\left(n-1\right)}{2}}$$

where *η*(*γ*) is the apparent viscosity at the shear rate *γ*, *η*_o_ is the zero-shear viscosity, *λ* is a time constant related to the onset of shear thinning, and n is the flow behavior index. Model fitting was performed in Microsoft Excel using the Solver add-in, minimizing the sum of squared residuals between the experimental data and the model predictions. Due to the high viscosity of the ionic liquid, only data obtained at temperatures above 298.15 K were reliable for fitting. Therefore, for comparison with the ethyl-substituted ionic liquid, data in the temperature range of 298.15 to 323.15 K are presented. Experimental viscosity as a function of shear rate for pure 1-carboxyethyl-3-butylimidazolium chloride measured at temperatures between 298.15 K and 323.15 K is presented in Figure S9.

Electrical conductivity measurements of the pure ionic liquids were performed using a Pyrex conductivity cell equipped with platinum electrodes, over the temperature range from *T* = 293.15 to 323.15 K. A Jenco 3107 conductivity meter (Jenco Instruments Inc., San Diego, CA, USA) operating with a direct current signal was used for the measurements. The cell was calibrated using a standard 0.1000 mol·dm^−3^ KCl solution, yielding a cell constant of 1.0353 cm^−1^, which was periodically verified to ensure measurement consistency. To minimize self-heating effects and electrode polarization, at least ten consecutive readings were taken at 5 s intervals. The reported conductivity values represent the average of three measurements, with a relative standard uncertainty estimated to be below 1.5%.

The thermal properties of the synthesized ionic liquids were analyzed using a simultaneous Mettler Toledo TGA/DSC instrument (Mettler Toledo, Schwerzenbach, Switzerland) in a temperature range from 298.15 to 773.15 K. The heating rate was 10 K·min^−1^. During the measurement, the furnace was purged with nitrogen at a flow rate of 50 cm^3^·min^−1^. To provide an inert atmosphere during the measurement, the sample was kept at 298.15 K for 10 min at the beginning of the measurement and purged with nitrogen, after which heating began. For weighing, 150 µL platinum crucibles were used, and the initial mass of the samples was approximately 7 mg. The blank curve was subtracted for all measurements. Temperature and enthalpy calibration were performed with high-purity indium and zinc. Both standards were supplied by Mettler Toledo.

The differential scanning calorimetry (DSC) measurements of the ionic liquids were recorded using a DSC1 instrument (Mettler Toledo, Schwerzenbach, Switzerland) under an atmosphere of nitrogen (flow rate 50 cm^3^·min^−1^). The initial mass used for the DSC measurement was approximately 8 mg. Samples were carefully weighed into aluminium crucibles using a Mettler Toledo MX5 microbalance and hermetically sealed with an aluminium lid. After being inserted into a DSC cell, the cell was cooled from room temperature to 193.15 K with a cooling rate of 2 K·min^−1^, stayed at this temperature for 10 min, and then heated to 423.15 K with a heating rate of 5 K·min^−1^. For this temperature range, temperature and enthalpy were calibrated using *n*-octane (reference substance for gas chromatography, 99.3%, supplied by Merck, Darmstadt, Germany) and Milli-Q water.

### 3.3. Atomistic and Machine Learning Modeling

The structures of the synthesized ionic liquids were subjected to the global geometry optimization and ensemble generator (GOAT) workflow [29,30,31,32] to obtain their global minimum conformations, which served as the basis for subsequent atomistic calculations using DFT optimizations. The GOAT procedure was performed using the GFN-FF force field, developed by Grimme and co-workers [33,34,35,36,37]. The GOAT global minima were reoptimized at the r^2^SCAN-3c [38] level of theory to obtain improved ground-state geometries and to compute quantum-molecular descriptors, including ion-pair binding energies, a widely used atomic-level descriptor in the study of ionic liquids. All DFT calculations have been performed with ORCA6.0.1 [32,39,40,41,42,43,44,45,46] code, while SAPT2 calculations were performed with the PSI4 [47,48,49] code. In the case of SAPT2 calculations, a jun-cc-PVDZ basis set was applied, while the input file was generated using the “Online input generator for PSI4” within the Atomistica Online 2025 online application. For density estimation, the IonIL-IM-D1 machine learning model was applied [26], which relies on three simple molecular features and therefore does not require prior geometry optimization. These density predictions were carried out using the “Online IL Density Predictor” tool, which employs the IonIL-IM-D1 model, part of the Atomistica Online 2025 online application, freely available for academic purposes at https://atomistica.online (accessed on 15 January 2026) [26,50,51]. The IonIL-IM-D1 model was developed and validated using a dataset of 434 imidazolium-based ionic liquids with experimentally determined densities at 298 K, employing an 80:20 training/test split, and is based on a support vector regression (SVR) algorithm. It relies exclusively on three readily accessible molecular descriptors (molecular weight, number of atoms, and ALogP), which can be obtained without quantum-mechanical calculations or geometry optimization, making the model particularly suitable for rapid prediction and high-throughput screening of ionic liquids. Importantly, the ionic liquids investigated in the present work are represented within the descriptor domain of the IonIL-IM-D1 model, as their molecular descriptor values fall within the ranges covered by the training dataset.

## 4. Conclusions

In this study, a series of novel carboxy-functionalized imidazolium chloride ionic liquids was synthesized and investigated through an integrated experimental and computational strategy to overcome characterization challenges. Key findings can be summarized as follows:

1. Electrostatic interactions are a primary determinant of bulk properties, as revealed by SAPT2 energy decomposition. However, the correlation with viscosity is qualitative and based on limited experimental data; steric and dispersive effects can modulate the electrostatic-driven trends, as seen in the butyl-substituted ILs. It should be noted that the SAPT2 energy decomposition analysis was performed for isolated gas-phase ion pairs; therefore, the observed relationships with bulk physicochemical properties should be interpreted as molecular-level correlations rather than direct simulations of the condensed phase.

2. The observed correlation between higher predicted/measured densities, higher viscosities, and increased thermal decomposition temperatures is consistent with tighter ion–ion associations driven by electrostatics, although direct investigation of ion packing and free volume was not performed in this study.

3. Alkyl chain length modulates properties through competing effects. While elongation of the carboxy-alkyl chain (e.g., from methyl to ethyl) reduced electrostatic cohesion and viscosity, elongation of the N-alkyl chain (e.g., from ethyl to butyl) introduced steric and dispersive effects that could increase viscosity, demonstrating a nuanced structure–property relationship.

4. The IonIL-IM-D1 model demonstrated <1% error on the compound for which experimental density could be measured ([C_2_COOHeim][Cl]). For other compounds, where direct measurement was not possible, predictions represent estimates requiring experimental validation.

Collectively, these results demonstrate that an integrated approach combining targeted experimentation with computational and ML methods provides a powerful framework for elucidating structure–property relationships in challenging ionic liquid systems. The insights and predictive framework established here lay a foundation for the rational design of ILs with tailored viscosities, densities, and thermal stability.

## Figures and Tables

**Table 1:** The total ion-pair binding energies (IPBEs) values of the studied ionic liquids.

Ionic Liquid	IPBE (kcal·mol^−1^)
[C_1_COOHmim][Cl]	−104.02
[C_2_COOHmim][Cl]	−98.04
[C_1_COOHeim][Cl]	−103.90
[C_2_COOHeim][Cl]	−102.12
[C_2_COOHbim][Cl]	−102.54

**Table 2:** Symmetry-adapted perturbation theory (SAPT2) interaction energy and its components (in kcal·mol^−1^): electrostatic (EL), exchange (EX), induction (I), and dispersion (D).

System	EL	EX	I	D	Total SAPT2
[C_1_COOHmim][Cl]	−121.40	45.07	−24.51	−4.33	−105.17
[C_2_COOHmim][Cl]	−120.22	45.16	−20.76	−4.96	−100.76
[C_1_COOHeim][Cl]	−121.22	45.99	−25.09	−4.63	−104.95
[C_2_COOHeim][Cl]	−117.91	45.07	−23.67	−4.73	−101.23
[C_2_COOHbim][Cl]	−116.95	46.77	−25.98	−4.88	−101.04

**Table 3:** Molecular weight, number of atoms, AlogP, experimental density, and IonIL-IM-D1 model for density prediction of the studied ILs.

Ionic Liquids	Molecular Weight	Number of Atoms	AlogP	Experimental Density (g/cm^3^)	IonIL-IM-D1 Density (g·cm^−3^)
[C_1_COOHmim][Cl]	177.611800	20	−0.985200	/	1.26609
[C_2_COOHmim][Cl]	191.638890	23	−0.737000	/	1.25275
[C_1_COOHeim][Cl]	191.638890	23	−0.636400	/	1.25160
[C_2_COOHeim][Cl]	205.665980	26	−0.388200	1.23611	1.23519
[C_2_COOHbim][Cl]	233.720160	32	0.591700	/	1.18236

**Table 4:** Correlation of the physicochemical features of the studied ILs with their thermal properties.

Ionic Liquid	*d* at 298.15 K (g·cm^−3^)	η at 298.15 K (Pa·s)	*T*_onset_ (°C)	*T*_mp_ (°C)	*T*_g_ (°C)
[C_1_COOHmim][Cl]	1.26609 (pred.)	solid at RT	250	178	N/A
[C_2_COOHmim][Cl]	1.25275 (pred.)	instr. limitation	196		−51
[C_1_COOHeim][Cl]	1.25160 (pred.)	solid at RT	252	167	N/A
[C_2_COOHeim][Cl]	1.23611 (exp.)	25.784	198		−46
[C_2_COOHbim][Cl]	1.18236 (pred.)	36.107	205		−47

**Table 5:** Proposed ILs for screening with the IonIL-IM-D1 model for density.

Ionic Liquids	Name of Ionic Liquids	Abbreviation
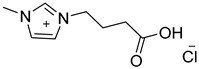	1-carboxypropyl-3-methylimidazolium chloride	[C_3_COOHmim][Cl]
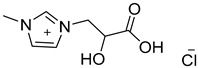	1-(2-hydroxy-2-carboxyethyl)-3-methylimidazolium chloride	[C_2_(OH)COOHmim][Cl]
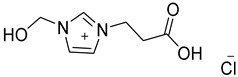	1-carboxyethyl-3-hydroxymethylimidazolium chloride	[C_2_COOHm(OH)im][Cl]
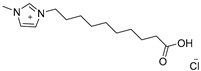	1-carboxynonyl-3-methylimidazolium chloride	[C_9_COOHmim][Cl]
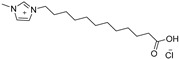	1-carboxyundecyl-3-methylimidazolium chloride	[C_11_COOHmim][Cl]

**Table 6:** IPBE values of the proposed ionic liquids.

Ionic Liquid	IPBE (kcal·mol^−1^)
[C_3_COOHmim][Cl]	−102.03
[C_2_(OH)COOHmim][Cl]	−102.95
[C_2_COOHm(OH)im][Cl]	−112.64
[C_9_COOHmim][Cl]	−96.02
[C_11_COOHmim][Cl]	−95.52

**Table 7:** SAPT2 interaction energy and its components (in kcal·mol^−1^) of postulated ILs.

Ionic Liquid	EL	EX	I	D	Total SAPT2
[C_3_COOHmim][Cl]	−116.69	45.73	−25.47	−4.50	−100.93
[C_2_(OH)COOHmim][Cl]	−125.93	51.18	−22.60	−5.60	−102.95
[C_2_COOHm(OH)im][Cl]	−131.36	47.66	−23.04	−4.95	−111.69
[C_9_COOHmim][Cl]	−98.16	40.46	−24.25	−4.40	−86.35
[C_11_COOHmim][Cl]	−98.14	40.44	−23.47	−4.82	−85.99

**Table 8:** Predicted densities of proposed ILs by the IonIL-IM-D1 model.

Ionic Liquid	Molecular Weight	Number of Atoms	AlogP	IonIL-IM-D1 Density (g·cm^−3^)
[C_3_COOHmim][Cl]	205.665980	26	−0.415800	1.235811
[C_2_(OH)COOHmim][Cl]	207.638290	24	−1.389200	1.294617
[C_2_COOHm(OH)im][Cl]	207.638290	24	−1.294100	1.295845
[C_9_COOHmim][Cl]	289.828520	44	2.321400	1.099632
[C_11_COOHmim][Cl]	317.882700	50	3.233800	1.072722

**Table 9:** Chemical structures, states at room temperature (RT), chemical names, abbreviations, and purification methods of synthesized ionic liquids.

Ionic Liquid	Chemical Name	Abbreviation	State at RT	Purification Method	Yield (%)
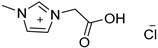	1-carboxymethyl-3-methylimidazolium chloride	[C_1_COOHmim][Cl]	solid	Solid-liquid separation	85%
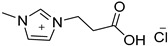	1-carboxyethyl-3-methylimidazolium chloride	[C_2_COOHmim][Cl]	liquid	Liquid-liquid extraction	80%
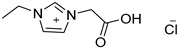	1-carboxymethyl-3-ethylimidazolium chloride	[C_1_COOHeim][Cl]	solid	Solid-liquid separation	90%
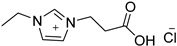	1-carboxyethyl-3-ethylimidazolium chloride	[C_2_COOHeim][Cl]	liquid	Liquid-liquid extraction	86%
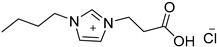	1-carboxyethyl-3-butylimidazolium chloride	[C_2_COOHbim][Cl]	liquid	Liquid-liquid extraction	83%

## Data Availability

The original contributions presented in this study are included in the article/Supplementary Materials. Further inquiries can be directed to the corresponding author.

## Supplementary Materials

The following supporting information can be downloaded at: https://www.mdpi.com/article/10.3390/molecules31142495/s1.
