# Supplementary material for: Predicting Density and Elucidating the Thermodynamic Drivers of Viscosity in Carboxy-Functionalized Imidazolium Ionic Liquids
Source: Molecules. 2026 Jul 17;31(14):2495. doi: 10.3390/molecules31142495 (PMC13415305; doi:10.3390/molecules31142495)
Supplement: Supplementary file 1 [file molecules-31-02495-s001.zip › molecules-4385295-supplementary.pdf]

Supplementary Material

Predicting Density and Elucidating the Thermodynamic Drivers of Viscosity in Carboxy-Functionalized Imidazolium Ionic Liquids

Nikolett Cakó Bagány [1], Sanja Armaković [1], Stevan Armaković [1], Bojan Šarac [2], Sanja Belić [1], Romana Cerc-Korošec [2], Marija Bešter-Rogač [2] and Slobodan Gadžurić [1,*]

[1] Faculty of Science, University of Novi Sad, Trg Dositeja Obradovića 3, 21000 Novi Sad, Serbia; nikolet.cakobaganj@dh.uns.ac.rs (N.C.B.); sanja.armakovic@dh.uns.ac.rs (S.A.); stevan.armakovic@df.uns.ac.rs (S.A.); sanja.belic@dh.uns.ac.rs (S.B.)

[2] Faculty of Chemistry and Chemical Technology, University of Ljubljana, Večna pot 113, 1000 Ljubljana, Slovenia; bojan.sarac@fkkt.uni-lj.si (B.Š.); romana.cerc-korosec@fkkt.uni-lj.si (R.C.-K.); marija.bester@fkkt.uni-lj.si (M.B.-R.)

* Correspondence: slobodan.gadzuric@dh.uns.ac.rs; Tel.: +381-21-485-2744; Fax: +381-21-454-065

Table S1. Density (d), thermal expansion coefficient (α_p), specific conductivity (κ) and molar conductivity (Λ) values for [C_2COOHeim][Cl] in the temperature range from T = (293.15 to 323.15) K

T (K)	d ($g \cdot cm^{-3}$)	$\alpha_p \cdot 10^4$ (K^{-1})	κ ($mS \cdot cm^{-1}$)	Λ ($S \cdot cm^2 \cdot mol^{-1}$)
293.15	1.23919	5.30	-	-
298.15	1.23611	5.31	0.048	0.0080
303.15	1.23292	5.32	0.080	0.0133
308.15	1.22956	5.34	0.122	0.0204
313.15	1.22613	5.35	0.160	0.0267
318.15	1.22294	5.37	0.225	0.0376
323.15	1.21960	5.38	0.305	0.0512

Table S2. Estimated viscosity values (η) of selected ionic liquids

T (K)	η (Pa·s) [C_2COOHeim][Cl]	η (Pa·s) [C_2COOHbim][Cl]
293.15	-	74.797
298.15	25.784	36.107
303.15	14.429	20.511
308.15	8.004	15.291
313.15	5.035	12.890
318.15	3.389	9.526
323.15	2.457	7.632

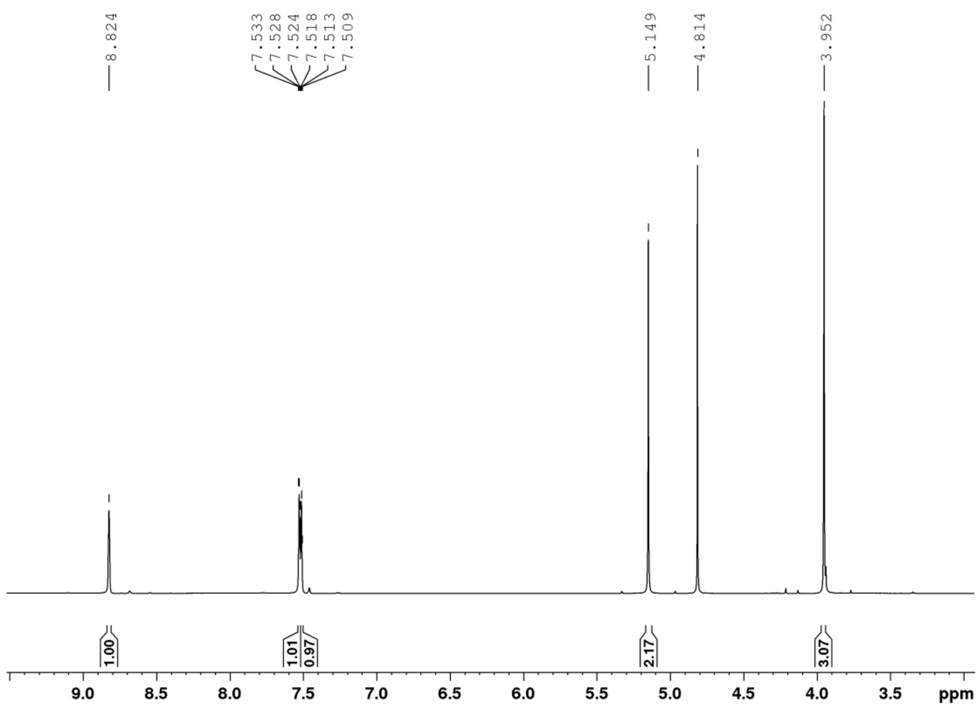

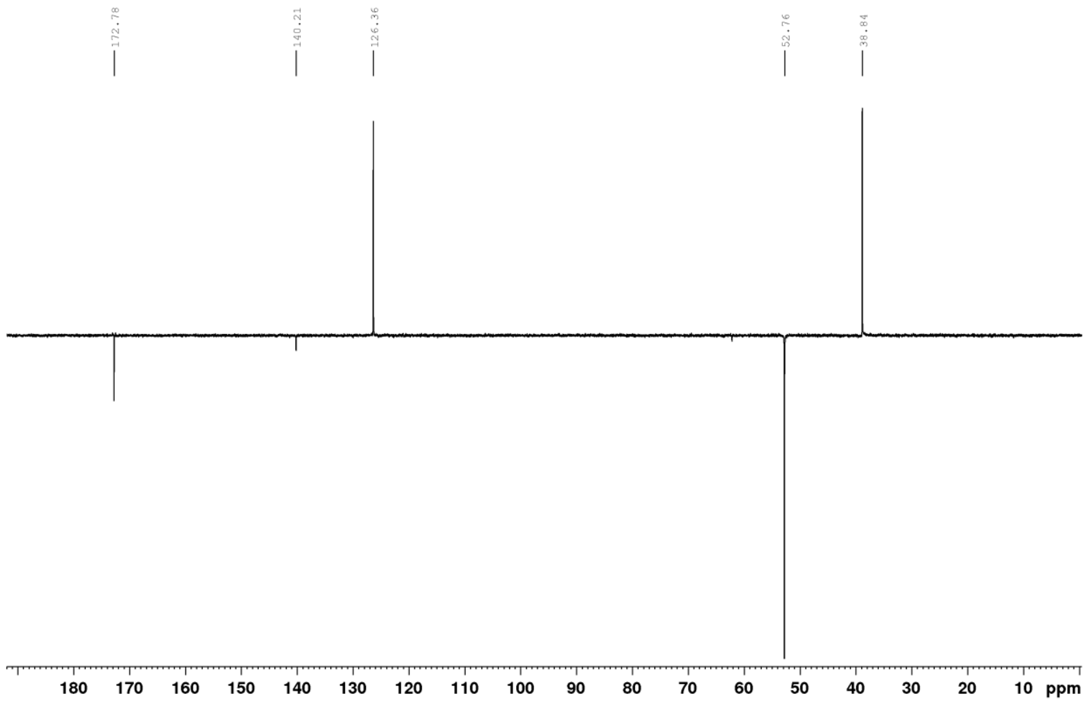

Figure S1. ^{1}H NMR and ^{13}C NMR spectra of [C_1COOHmim][Cl]

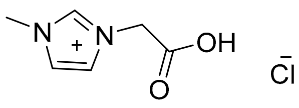

^{1}H NMR assignation [δ, ppm]: 3.95 (s, 3H, CH_3); 5.15 (s, 2H, CH_2); 7.52 и 7.53 (2 × t, 2 × 1H, *J* = 1.7 Hz, NC*H*C*H*N); 8.82 (bs, 1H, NC*H*N).

^{13}C NMR assignation [δ, ppm]: 38.84 (CH_3); 52.76 (CH_2); 126.36 (2 × C, N*C*H*C*HN); 140.21 (N*C*HN); 172.78 (C=O, *C*OOH).

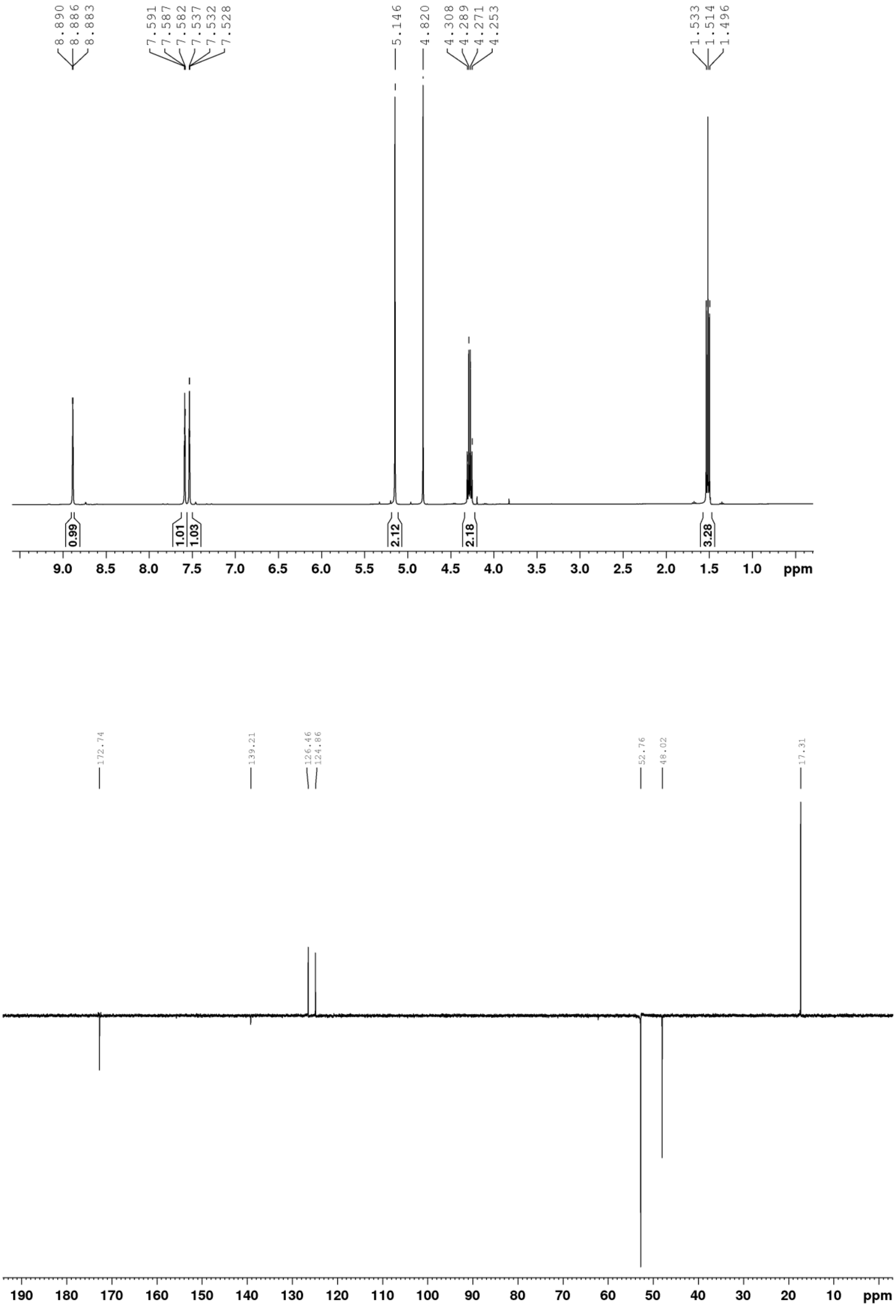

Figure S2. ^{1}H NMR and ^{13}C NMR spectra of [C_1COOHeim][Cl]

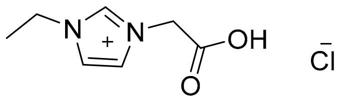

^{1}H NMR assignation [δ, ppm]: 1.52 (t, 3H, *J* = 7.4 Hz, CH_3CH_2N); 4.28 (q, 2H, *J* = 7.4 Hz, CH_3CH_2N); 5.15 (s, 2H, NCH_2COOH); 7.54 и 7.59 (2 × t, 2 × 1H, *J* = 1.8 Hz, NC*H*C*H*N); 8.89 (pseudo t, *J* = 1,7 Hz, 1H, NC*H*N).

^{13}C NMR assignation [δ, ppm]: 17.31 (CH_3CH_2N); 48.02 (CH_3CH_2N); 52.76 (NCH_2COOH); 124.86 и 126.46 (2 × C, NCH*C*HN); 139.21 (N*C*HN); 172.74 (C=O, *C*OOH).

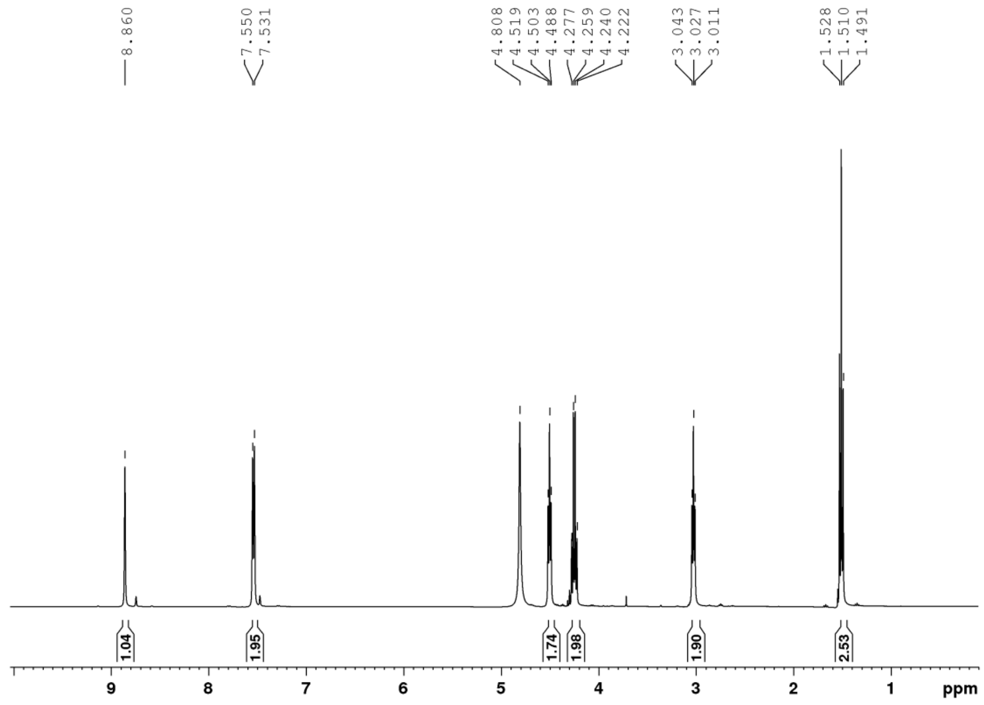

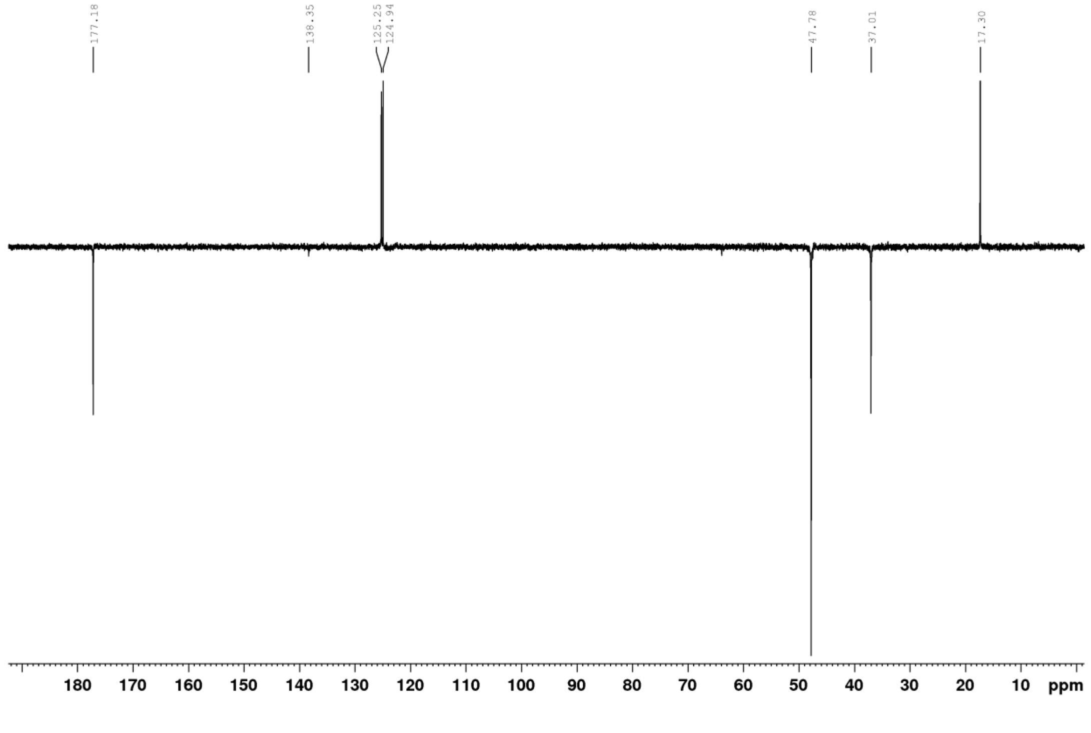

Figure S3. ^{1}H NMR and ^{13}C NMR spectra of [C_2COOHeim][Cl]

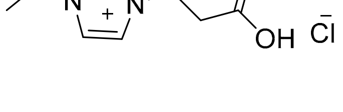

^{1}H NMR assignation [δ, ppm]: 1.52 (t, 3H, J = 7.4 Hz, CH_3CH_2N); 3.00 (t, 2H, J = 6.3 Hz, NCH_2CH_2COOH); 4.26 (q, 2H, J = 7.4 Hz, CH_3CH_2N); 4.50 (t, 2 H, J = 6.3 Hz, NCH_2CH_2COOH); 7.54 и 7.56 (2 × t, 2 × 1H, J = 1.9 Hz, NC*H*C*H*N); 8.86 (pseudo t, 1H, J = 1.7 Hz, NC*H*N).

^{13}C NMR assignation [δ, ppm]: 17.30 (CH_3CH_2N); 37.01 (NCH_2CH_2COOH); 47.78 (2 × C, NCH_2CH_2COOH и CH_3CH_2N); 124.94 i 125.25 (2 × C, N*C*H*C*HN); 138.35 (N*C*HN); 177.18 (C=O, *C*OOH).

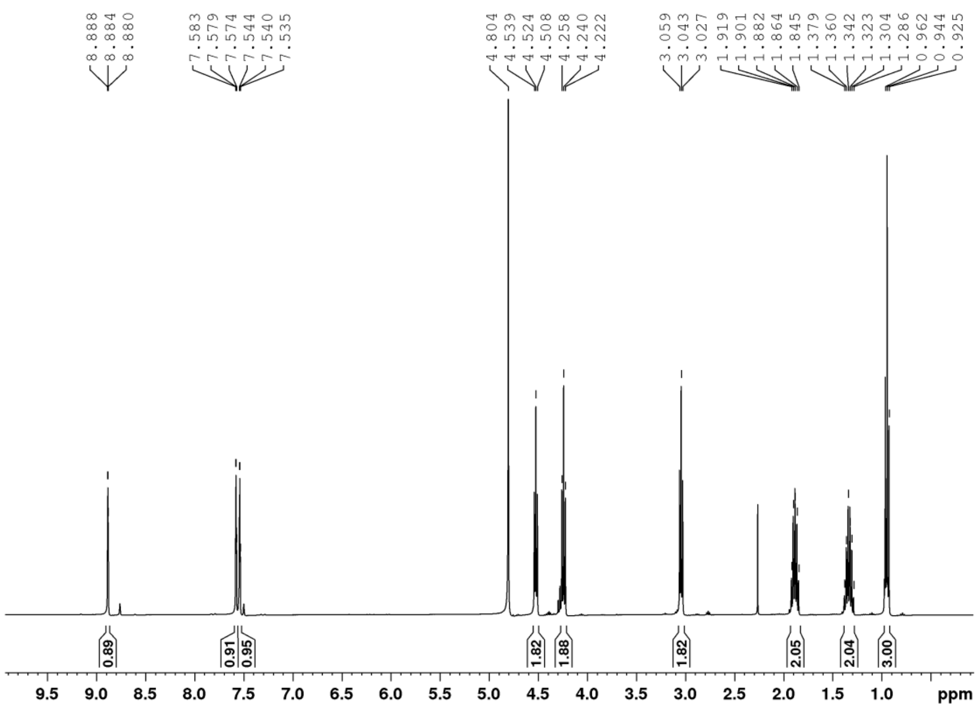

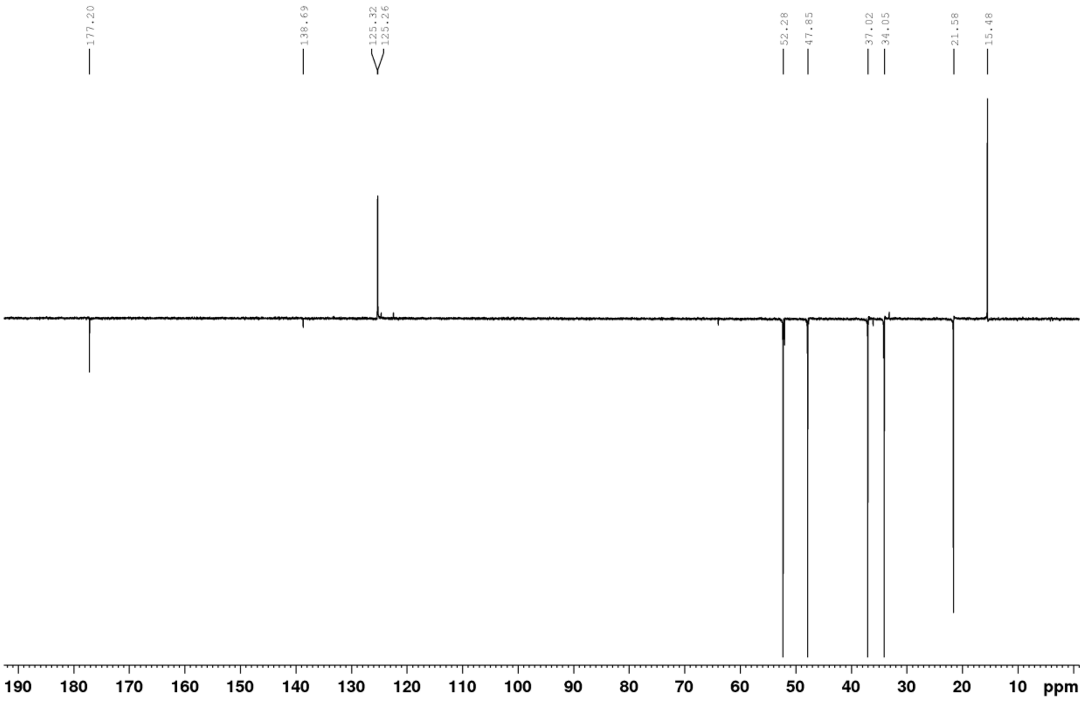

Figure S4. ^{1}H NMR and ^{13}C NMR spectra of [C2COOHbim][Cl]

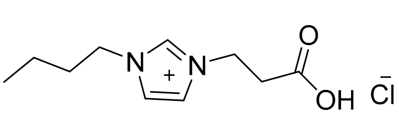

^{1}H NMR assignation [δ, ppm]: 0.95 (t, 3H, *J* = 7.4 Hz, C*H3*CH2CH2CH2N); 1.33 (sex, 2H, *J* = 7.6 Hz, CH3C*H2*CH2CH2N); 1.88 (qui, 2H, *J* = 7.3 Hz, CH3CH2C*H2*CH2N); 3.05 (t, 2H, *J* = 6.3 Hz, NCH2C*H2*COOH); 4.24 (t, 2H, *J* = 7.2 Hz, CH3CH2CH2C*H2*N); 4.53 (t, 2H, *J* = 6.3 Hz, NC*H2*CH2COOH); 7.54 и 7.58 (2 × t, 2 × 1H, *J* = 1.8 Hz, NC*H*C*H*N); 8.88 (pseudo t, *J* = 1.7 Hz, 1H, NC*H*N).

^{13}C NMR assignation [δ, ppm]: 15.48 (*C*H3CH2CH2CH2N); 21.58 (CH3*C*H2CH2CH2N); 34.05 (CH3CH2*C*H2CH2N); 37.02 (NCH2*C*H2COOH); 47.85 (N*C*H2CH2COOH); 52.28 (CH3CH2CH2*C*H2N); 125.26 и 125.32 (2 × C, N*C*H*C*HN), 138.69 (N*C*HN); 177.20 (C=O, *C*OOH).

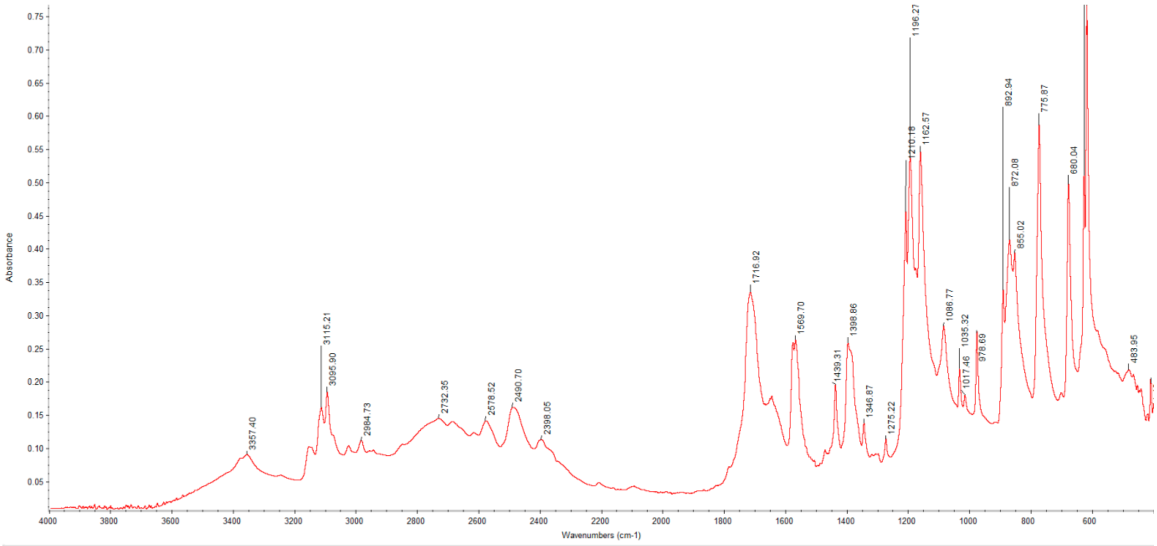

Figure S5. FTIR spectra of [C_1COOHmim][Cl]

IR (neat): 3357 (stretching ν O–H from –COOH); 3115 (symmetric stretching ν HC(4)/HC(5) of imidazolium ring); 2984, 2938 (asymmetric stretching ν CH_3 / CH_2); 1716 (stretching ν C=O from –COOH); 1569 (in-plane vibrations of imidazolium ring); 1275 (C–N stretching from imidazolium ring); 1035 (skeletal vibrations of imidazolium ring); 775 (in-plane ring bending δ CC); 680 (out-of-plane ring deformation).

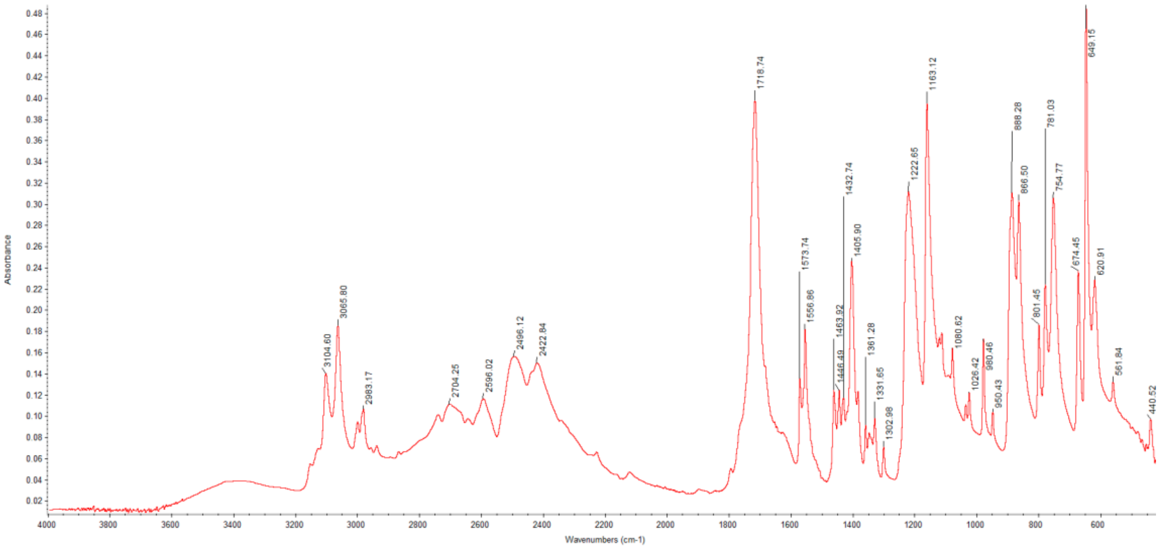

Figure S6. FTIR spectra of [C_1COOHeim][Cl]

IR (neat): 3065 (symmetric stretching ν HC(4)/HC(5) of imidazolium ring); 2983 (asymmetric stretching ν CH_3 / CH_2 from ethyl and carboxymethyl groups); 1718 (stretching ν C=O from –COOH); 1573 (in-plane stretching vibrations of imidazolium ring); 1456, 1432 (CH_2 and CH_3 bending); 1315 (C–N stretching); 1222 (C–O stretching from –COOH); 1103, 1062 (skeletal ring vibrations); 754 (in-plane bending δ CC); 674, 620 (ring deformation).

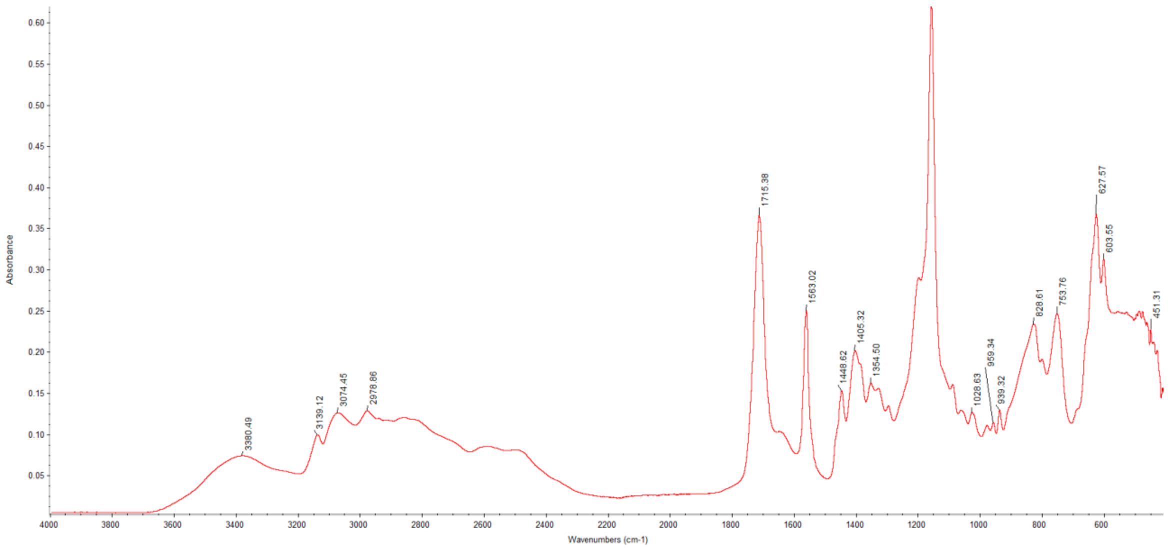

Figure S7. FTIR spectra of [C2COOHeim][Cl]

IR (neat): 3360 (stretching ν O–H from –COOH); 3091 (symmetric stretching ν HC(4)/HC(5) of imidazolium ring); 2978 (asymmetric stretching ν CH_3 / CH_2 from ethyl and carboxyethyl groups); 1715 (stretching ν C=O from –COOH); 1563 (in-plane stretching vibrations of imidazolium ring); 1448 (CH_2 / CH_3 bending); 1205 (scissoring vibration of –OH from –COOH); 1053 (skeletal vibrations of imidazolium ring); 763 (in-plane ring bending δ CC).

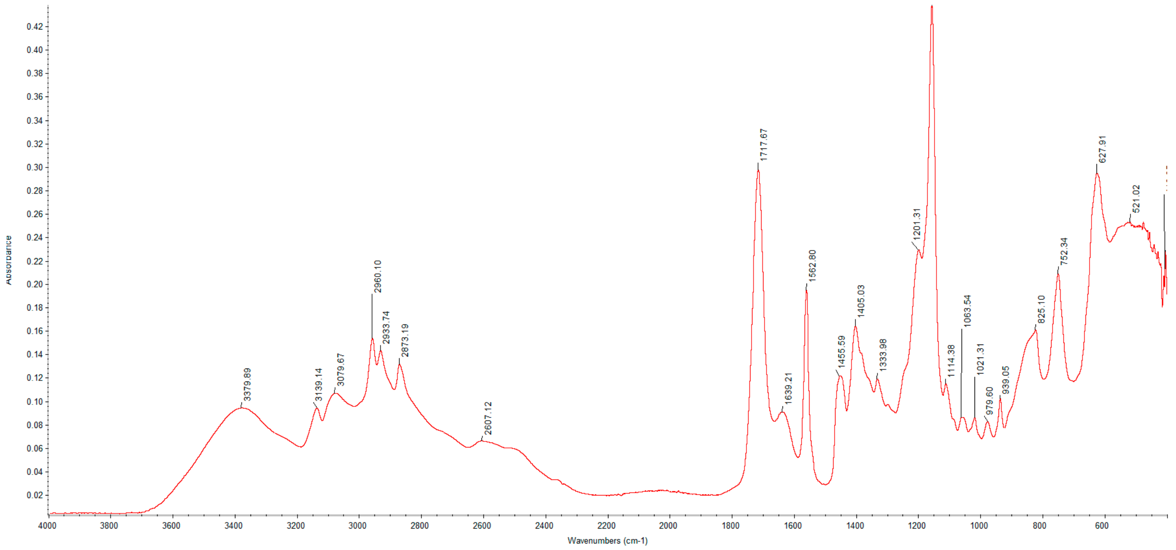

Figure S8. FTIR spectra of [C2COOHbim][Cl]

IR (neat): 3379 (stretching ν O–H from –COOH); 3079 (symmetric stretching ν HC(4)/HC(5) of imidazolium ring); 2933 (asymmetric stretching ν CH_3 / CH_2 from butyl and carboxyethyl groups); 1717 (stretching ν C=O from –COOH); 1638 (in-plane vibrations of imidazolium ring); 1562 (δ CH_2 / CH_3 bending); 1405 (C–N stretching and ring vibrations); 1201 (C–O stretching and ring deformations); 1021 (skeletal ring vibrations, C–O, and alkyl chain modes); 762 (in-plane bending δ CC).

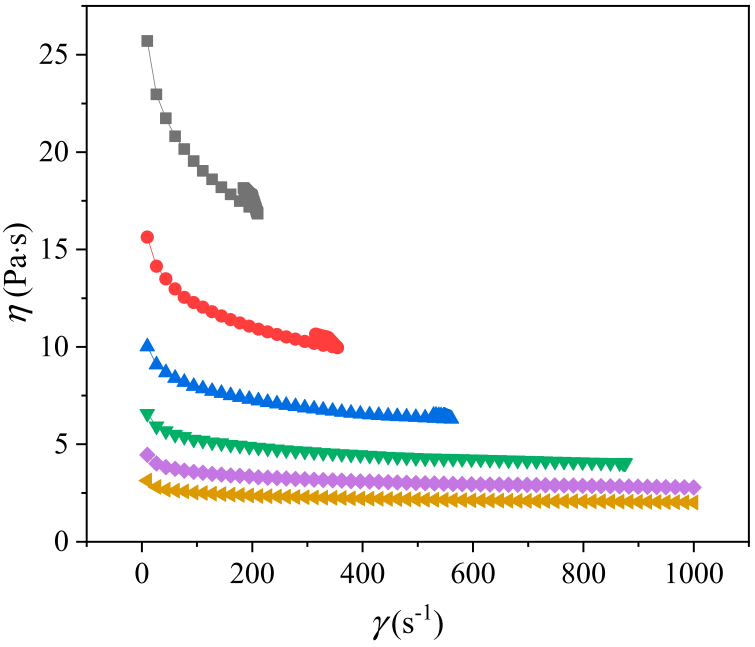

Figure S9. Experimental viscosity as a function of shear rate for pure 1-carboxyethyl-3-butylimidazolium chloride measured at temperatures between (298.15 and 323.15) K
